# Primary diffuse large B‐cell lymphoma presenting as acute appendicitis: A report of 2 cases and a literature review

**DOI:** 10.1002/ccr3.2653

**Published:** 2020-01-07

**Authors:** Maria Jose Fernandez Turizo, Mohamed A. Kharfan‐Dabaja, Muhamad Alhaj Moustafa, Ernesto Ayala, Liuyan Jiang, Ricardo Parrondo

**Affiliations:** ^1^ Division of Hematology‐Oncology Mayo Clinic Jacksonville FL USA

**Keywords:** diffuse large B‐cell lymphoma, non‐Hodgkin lymphoma, primary appendiceal lymphoma, primary gastrointestinal non‐Hodgkin lymphoma

## Abstract

Primary appendiceal lymphomas (PAL) are a type of primary gastrointestinal non‐Hodgkin lymphoma (PGINHL) with an incidence of <1%. There is considerable discordance with regard to the optimal management of PGINHL. We describe two cases of PAL, perform a literature review, and discuss the available evidence for optimal treatment.

## Graphical abstract

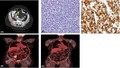

## 1 INTRODUCTION

Primary gastrointestinal non‐hodgkin lymphoma constitute some of the most common types of extra‐nodal non‐Hodgkin lymphomas, accounting for 30%‐40% of cases.^1^ However, these lymphomas are relatively rare, accounting for only 1%‐4% of all gastrointestinal (GI) malignancies.^2^ The definition of a primary GI lymphoma was first described by Dawson et al^3^ as a “predominantly gastrointestinal tract lesion, with or without spread to regional nodes, no involvement of the peripheral or mediastinal nodes, no involvement of the liver or spleen and a normal white cell count and differential”. Based on a population‐based registry, Gurney et al analyzed 1069 cases of PGINHL and reported that the most common site of PGINHL was gastric (43.3%), followed by small bowel (27.4%), large bowel (11.1%), and site unknown (16.1%). Most PGINHL cases in this series were high grade (44.5%), with 30.4% being low‐grade, 19.0% unclassified, and 6.1% T‐cell lymphomas. From 1986 to 1993, the incidence of PGINHL increased by 2.7% per year.^4^ PAL, defined as lymphomas limited to the appendix, are especially rare and comprise 0.015% of all gastrointestinal lymphoma cases.^5^ Most cases of PGINHL are treated with a multi‐modality approach including chemotherapy, surgical resection, radiotherapy, and immunotherapy for optimal management. Due to the emergent presentation of PAL which mimics acute appendicitis, most patients are treated with surgical resection followed by six cycles of rituximab, cyclophosphamide, doxorubicin, vincristine, and prednisone every 21 days (R‐CHOP 21). Here, we describe two cases of diffuse large B‐cell PAL treated at our institution and we performed a review of the literature on optimal treatment strategies for this rare disease.

## 2 CASE PRESENTATION

### 2.1 Case #1

**Figure 1:**
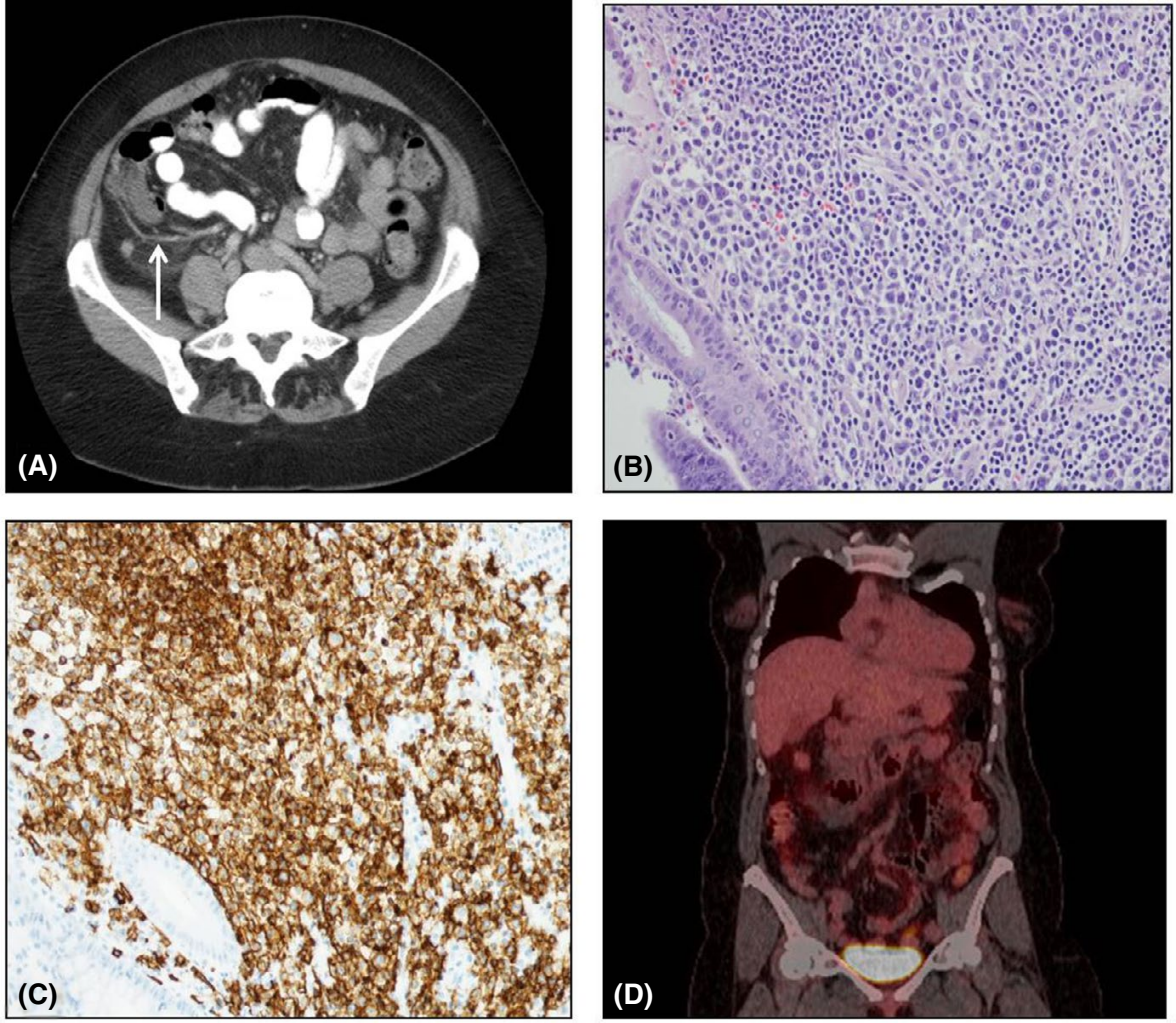
A, CT scan showing dilated appendix with nonspecific periappendiceal inflammatory changes (white arrow). B, 200× H&E stain of appendix tissue revealing large atypical mononucleated cells. C. 200× CD20 stain of appendix tissue. D, PET‐CT at diagnosis revealing no evidence of hypermetabolism outside of the appendix

A 57‐year‐old woman with a past history of major depressive disorder and immune thrombocytopenic purpura presented to the emergency department at our institution with a 3‐day history of 8‐10 daily episodes of watery diarrhea with an associated dull, persistent, and progressively worsening right lower quadrant (RLQ) abdominal pain. The patient was afebrile with a platelet count of 103 × 10^9^/L, normal hemoglobin and white blood cell count and a normal comprehensive metabolic panel. On physical examination, the patient had both RLQ tenderness to palpation and rebound tenderness and a positive Rovsing's sign. An abdominal CT scan was performed and demonstrated a dilated appendix with nonspecific periappendiceal inflammatory changes (Figure 1A). The patient was admitted to the hospital and taken to the operating room for presumed acute appendicitis. A laparoscopic appendectomy was performed. Surgical pathology of the appendix revealed an anaplastic variant of diffuse large B‐cell lymphoma (DLBCL). The appendix specimen had focal clusters of large atypical mononucleated cells that invaded through the muscularis propria (Figure 1B). The atypical cells were strongly positive for CD30, PAX‐5, and CD20 (Figure 1C); negative for Melan‐A, S‐100, pancytokeratin, CD15, and CD3. EBER in situ hybridization for EBV was negative. The neoplastic lymphocytes were strongly positive for MUM1, focally positive for BCL2; negative for BCL6 and CD10. The immunoprofile was consistent of activated B‐cell phenotype. No rearrangement of MYC and no fusion of MYC and IGH gene regions were observed. A bone marrow biopsy showed no morphologic or phenotypic evidence of metastatic DLBCL. Flow cytometry showed only polytypic B lymphocytes. A PET‐CT revealed no areas of suspicious hypermetabolism (Figure 1D). The patient was thus diagnosed with a Lugano Stage I primary DLBCL of the appendix. Her revised international prognostic index score (R‐IPI) was 0. The patient went on to complete six cycles R‐CHOP 21. The patient tolerated R‐CHOP relatively well and only developed grade 2 diarrhea as a side effect and without any hospitalizations, infections, delays in treatment, or transfusion requirements. Restaging CT of the chest, abdomen and pelvis did not reveal any evidence of lymphadenopathy. The patient remains disease‐free 1 year later.

### 2.2 Case #2

**Figure 2:**
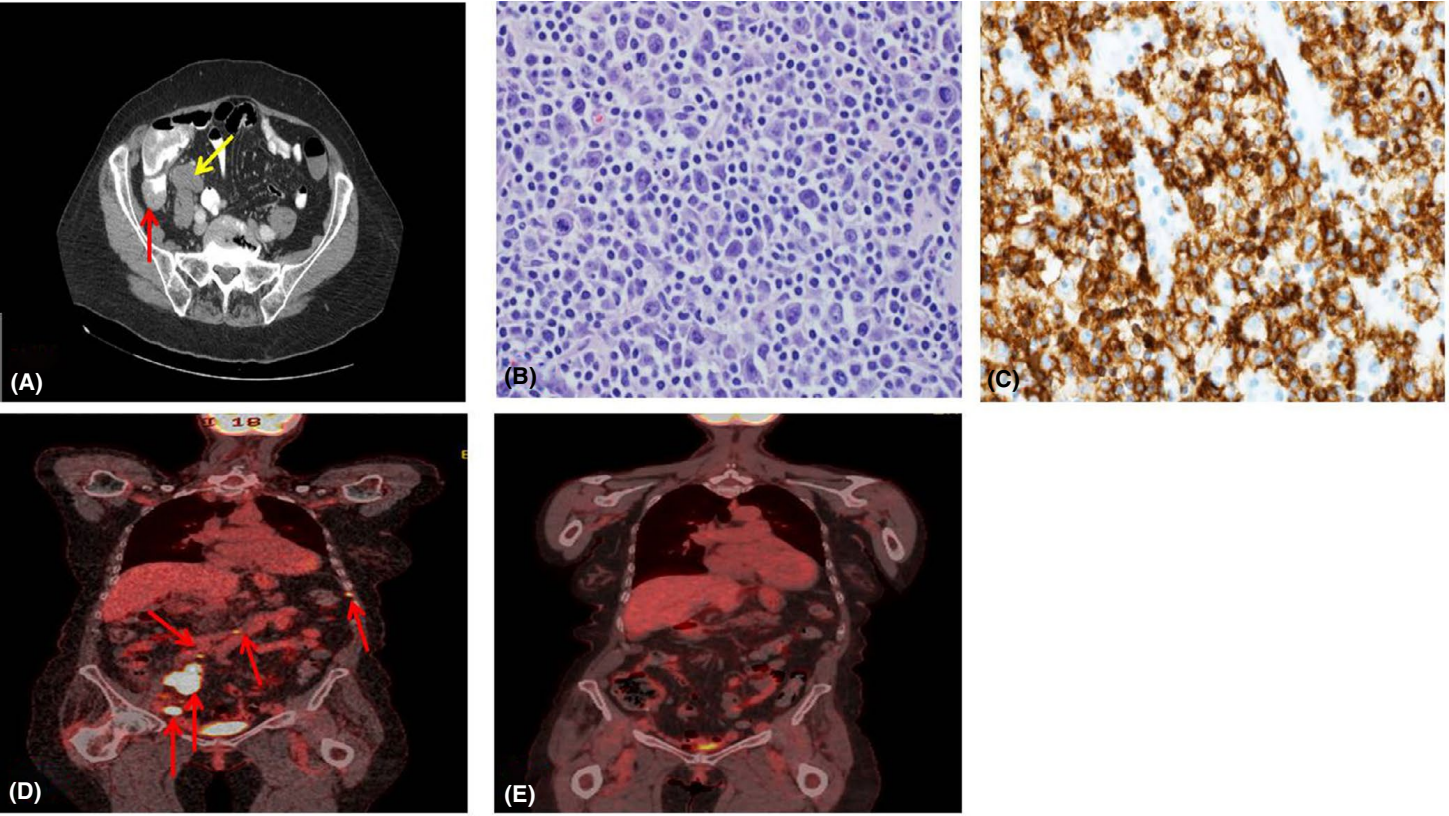
A, CT scan showing a dilated appendix with periappendiceal stranding (red arrow). In addition, there was a 3.8 × 2.1 cm mass in close vicinity (yellow arrow). B, 400× H&E stain of appendix tissue revealing large atypical mononucleated cells. C, 400× CD20 stain of appendix tissue. D, PET‐CT at diagnosis revealing a hypermetabolic intercostal focus between the left seventh and eight ribs, hypermetabolic aortocaval lymph nodes, and hypermetabolic right iliac lymph nodes (red arrows). E, PET‐CT following six cycles of R‐CHOP showing resolution of hypermetabolic areas

A 79‐year‐old woman with a history of essential hypertension presented to an outside hospital emergency department with a 1‐week history of constant, dull, and progressively worsening RLQ abdominal pain. She reported an unintentional seven pound weight loss over the preceding month as well as decreased appetite. She denied fevers or night sweats. Physical exam revealed both RLQ tenderness to palpation and rebound tenderness. She had a normal complete blood count and comprehensive metabolic panel. A CT of the abdomen was performed which showed a dilated appendix with periappendiceal stranding. In addition, there was a 3.8 × 2.1 cm mass in close vicinity (Figure 2A). She subsequently underwent a laparoscopic appendectomy and final pathology revealed a DLBCL (Figure 2B). Ki‐67 staining was 90%. The neoplastic lymphocytes were strongly positive for CD20 (Figure 2C), PAX5, and Bcl‐6; negative for CD56, S‐100, pancytokeratin, CD30, and CD3. MUM1 was positive in <20% of neoplastic cells. The immunoprofile was consistent with germinal center phenotype. No rearrangement of MYC and no fusion of MYC and IGH gene regions were observed. A bone marrow biopsy showed no morphologic or phenotypic evidence of metastatic DLBCL. Flow cytometry showed only polytypic B lymphocytes. A PET‐CT revealed a hypermetabolic left supraclavicular node, a hypermetabolic intercostal focus between the left seventh and eight ribs, hypermetabolic aortocaval lymph nodes, and hypermetabolic right iliac lymph nodes (Figure 2D). The patient was diagnosed with a Lugano Stage III primary DLBCL of the appendix. Her R‐IPI was 2. She received six cycles of R‐CHOP 21. R‐CHOP was relatively well tolerated with only grade 2 fatigue and grade 3 anemia. She was never hospitalized, she did not develop any infections, and there were no delays in treatment. An end of treatment, PET‐CT revealed a Deauville score of 1 (Figure 2E).

## 3 DISCUSSION

Cancers of the appendix are rare, and most are found incidentally on appendectomies performed for presumed appendicitis. PAL, which fall under the category of PGINHL, constitute a rare group of lymphomas representing <1% of NHL. We described two cases of patients with PAL who were treated with surgical resection and six cycles of R‐CHOP 21 and obtained a complete response. There are limited prospective trial data on optimal management of PGINHL, and most data are derived from retrospective studies. Furthermore, the optional management of PAL is based on retrospective studies or small nonrandomized prospective studies in which the majority had primary gastric or primary colonic lymphomas (Table 1).

**Table 1:** Studies describing treatment for PGINHL

Study	Type of NHL	Study type	n	Treatment arms	PFS	*P*	OS	*P*	Outcome
Kim et al^16^	Intestinal DLBCL	Retrospective	345	Resection followed by CHOP/R‐CHOP	82%^a^ 52%^b^ (3 yr)	<.001 .518	91%^a^ 58%^b^ (3 y)	<.001 .303	Resection plus chemo improves survival in lugano stage I/II DLBCL.
				CHOP/R‐CHOP alone	52%^a^ 34%^b^ (3 y)		62%^a^ 44%^b^ (3 y)		
Lai et al^17^	Colonic lymphoma	Retrospective	29	Resection followed by chemotherapy	NR	NR	75.5% (3 y)	.035	Resection plus chemo improves survival.
				Chemotherapy alone	NR		28.6% (3 y)		
Lee HS et al^18^	Intestinal DLBCL	Retrospective	76	Resection followed by R‐CHOP	92.2% (3 y)	.009	94.2% (3 y)	.049	Resection plus chemo improves survival.
				R‐CHOP alone	74.8% (3 y)		80.7% (3 y)		
Tang TC et al^19^	Colonic DLBCL	Retrospective	74	Resection followed by CHOP/COP	NR^c^ NR^d^	.567 .000	NR^c^ NR^d^	.389 .020	Resection plus COP but not CHOP chemo improves survival
				CHOP/COP alone	NR NR		NR NR		
Willich NA et al^9^	Gastric lymphoma	Prospective	257	Resection followed by CHOP x4 + EF RX if Stage I or CHOPx6 + IF RX if Stage II‐IV^e^	NR	NS	NR	NS	No survival differences between resection and chemo‐RT and chemo‐RT alone
				CHOP x4 + EF RX if Stage I or CHOPx6 + IF RX if Stage II‐IV^e^	NR		NR		
Aviles A et al^10^	Gastric DLBCL	Prospective	589	Resection	28% (10 y)	<.001	54% (10 y)	<.001	No survival differences between resection and resection + chemo
				Resection + RT	23% (10 y)		53% (10 y)		
				Resection + CHOP	82% (10 y)	NS	91% (10 y)	NS	
				CHOP	92% (10 y)		96% (10 y)		
Kim SJ et al^20^	Intestinal NHL	Retrospective	581	Resection	NR	NR	77% (5 y)	<.001	Survival benefit for resection in B‐cell but not T‐cell lymphomas
				No resection	NR		57% (5 y)		
Popescu RA et al^11^	Gastric NHL	Prospective	37	Chemo	62% (5 y)	NR	67% (5 y)	NR	No survival benefit for resection + chemo compared to chemo
				Resection + chemo	85% (5 y)		60% (5 y)		
Binn M et al^12^	Gastric DLBCL	Prospective	58	Resection + Chemo	91.6% (5 y)	.187	91.1% (5 y)	.303	No survival differences between resection + chemo and chemo alone
				Chemo	85.9% (5 y)		90.5% (5 y)		
Koch P et al^13^	Gastric lymphoma	Prospective	393	Resection + Chemo and/or RT	83.2% (3.5 y)	NS	86% (3.5 y)	NS	No survival differences between resection + chemo/RT or chemo/RT alone
				Chemo and/or RT	86% (3.5 y)		90.5% (3.5 y)		
Ayub A at al^6^	Primary appendiceal lymphoma	Retrospective	116	Appendectomy/partial colectomy	NR		12.3 y	.501	No survival differences based on extent of surgical resection
				Right hemicolectomy or greater			13 y		
Fischbach W et al^21^	Early stage gastric Lymphoma	Prospective	236	Resection followed by CHOP^α^	NR		88% (1.5 y)	<.001	Survival benefit for surgical resection of high grade gastric lymphoma
				CHOP + RT^α^	NR		53% (1.5 y)		
Shannon EM, et al^7^	PGINHL	Retrospective	16,129	Resection	NR		43.6% (5 y)	<.001	In multivariate analysis, resection did not improve overall survival (HR 1.05, 95% CI 0.96‐1.15, *P* = .298)
				No resection	NR		38.4% (5 y)		
Gobbi et al^14^	PGINHL	Prospective	154	Resection	NR	NR	NR	NS	Resection does not improve survival
				No resection	NR		NR		

Abbreviations: EF RX, extended field radiotherapy; IF RX, involved field radiotherapy; NR, not reported; NS, not significant.

a Lugano I/II.

b Lugano IV.

c CHOP.

d COP.

e High grade lymphoma.

In a retrospective analysis of 116 patients with PAL, Ayub et al showed that the mean age of diagnosis was 48 years, the population primarily affected was white males, the most common histology was DLBCL (34.5%) followed by Burkitt lymphoma (25.9%), the median overall survival was 185 months with a 5‐year survival rate of 67%, and right hemicolectomy conferred no survival benefit over appendectomy and/or partial colectomy.^6^ In a retrospective analysis of 16 129 patients with PGINHL (of which 0.6% were PAL), the most common histologies were DLBCL (63%), follicular (10.5%), mantle cell (2.5%), Burkitt (0.5%), and enteropathy‐associated T cell (EATL) (0.5%).^7^ Patients with PAL had the longest median survival at 45 months (*P* < .0001).^7^ Median survival differed by tumor histology; 20, 51, 25, 10, and 5 months for DLBCL, follicular, mantle cell, Burkitt, and EATL, respectively.^7^ Patients who underwent surgery had a median OS of 39 months compared to only 16 months in whom surgery was not recommended (*P* < .0001). Patients who underwent radiotherapy had significantly better median OS (40 months vs 22 months, *P* < .0001).^7^ Those who received surgery and radiation therapy had a significantly greater median survival of 69 months compared to 36 months for those who underwent surgery alone and 21.5 months for those who received radiotherapy alone (*P* < .0001).^7^ Data regarding chemotherapy administration were not available for analysis. However, in multivariate analysis, surgical resection was not associated with improved survival (HR 1.05; 95% CI 0.96‐1.15).^7^ The superior survival of patients with B‐cell compared to T‐cell PGINHL has been confirmed in other studies.^8^ In a prospective study of 56 patients with PGINHL, 2‐year OS rate for T‐cell lymphomas was 28% compared to 94% for B‐cell lymphomas (*P* < .0001).^8^

The role of surgery and surgery plus chemotherapy and/or radiation therapy in PGINHL is an area of ongoing debate. Several studies have shown no differences in survival for patients treated with chemotherapy and/or radiation therapy alone vs surgery plus chemotherapy and/or radiation therapy.^7^, ^9^, ^10^, ^11^, ^12^, ^13^, ^14^ A meta‐analysis of five studies containing a total of 701 patients with PGINHL revealed no differences in OS at 10 years between patients treated with chemotherapy and/or radiation therapy compared to patients treated surgically (HR 0.61, 95% CI 0.26‐1.41, *P* = .25); however, patients treated with chemotherapy and/or radiation therapy alone had superior disease‐free survival (OR 0.17, 95% CI 0.08‐0.37, *P* < .00001).^15^

Several studies have shown a survival benefit for surgery and chemotherapy/radiation therapy compared to chemotherapy and/or radiation therapy alone.^16^, ^17^, ^18^, ^19^, ^20^, ^21^ The largest of these trials was a retrospective study of 345 patients with GI DLBCL.^16^ The study revealed that patients with Lugano stage I/II GI DLBCL who underwent resection followed by chemotherapy with CHOP or R‐CHOP had a lower relapse rate compared to those who received chemotherapy alone (15.3% vs 36.8%, *P* < .001).^16^ The 3‐year OS rate was 91% in the surgery plus chemotherapy group and 62% in the chemotherapy group alone (*P* < .001).^16^ There were no PFS or OS differences between surgery and chemotherapy/radiation therapy compared to chemotherapy and/or radiation therapy alone in patients with Lugano stage IV DLBCL.^16^ The population of patients with Lugano stage III DLBCL was too small for analysis. R‐CHOP resulted in a twofold 3‐year OS advantage compared to CHOP (59% vs 29% *P* = .0678) albeit not statistically significant.^16^ Interestingly, one of our patients had a CD30^+^ DLBCL. It has been reported that CD30 expression is a favorable prognostic factor in a cohort of 903 patients with de novo DLBCL.^22^ Patients with CD30^+^ DLBCL had a superior 5‐year OS (CD30^+^, 79% vs CD30^‐^, 50%; *P* = .001 and PFS (*P* = .003).^22^ The favorable outcome of CD30 + expression was maintained in both the germinal center and activated B‐cell subtypes.^22^

## 4 CONCLUSION

Optimal management of PGINHL has been determined mostly from retrospective and small prospective nonrandomized studies. Based on the largest of these studies by Kim et al, patients with PAL should be managed with surgical resection followed by R‐CHOP 21 for six cycles whether they have localized disease (Lugano stage I/II) or disseminated disease (Lugano Stage IV). Furthermore, most patients undergo surgical resection due to the inability to distinguish acute appendicitis from PAL on imaging. Large multicenter studies are needed to determine the optimal management of PGINHL and PAL in particular.

## CONFLICTS OF INTEREST

The authors do not have any relevant conflicts of interest to report for this work.

## AUTHORS' CONTRIBUTIONS

RDP and MJFT: wrote the manuscript; LJ: obtained the pathology images for the case; and EA, MAM, and MA.K‐D: edited and finalized the manuscript.
